# N-acetylcysteine reduces oxidative stress, nuclear factor-κB activity and cardiomyocyte apoptosis in heart failure

**DOI:** 10.3892/mmr.2014.2292

**Published:** 2014-06-02

**Authors:** XIAO-YAN WU, AN-YU LUO, YI-RONG ZHOU, JIANG-HUA REN

**Affiliations:** 1 Department of Cardiology, Zhongnan Hospital of Wuhan University, Wuhan, Hubei, P.R. China; 2 Hanyang Hospital Affiliated to Wuhan University of Science and Technology, Wuhan, Hubei, P.R. China; 3 Department of Pharmacology and Toxicology, Wright State University, Dayton, OH, USA

**Keywords:** N-acetylcysteine, nuclear factor κ B, heart failure, apoptosis, reactive oxygen species

## Abstract

The roles of oxidative stress on nuclear factor (NF)-κB activity and cardiomyocyte apoptosis during heart failure were examined using the antioxidant N-acetylcysteine (NAC). Heart failure was established in Japanese white rabbits with intravenous injections of doxorubicin, with ten rabbits serving as a control group. Of the rabbits with heart failure, 12 were not treated (HF group) and 13 received NAC (NAC group). Cardiac function was assessed using echocardiography and hemodynamic analysis. Myocardial cell apoptosis, apoptosis-related protein expression, NF-κBp65 expression and activity, total anti-oxidative capacity (tAOC), 8-iso-prostaglandin F2α (8-iso-PGF2α) expression and glutathione (GSH) expression levels were determined. In the HF group, reduced tAOC, GSH levels and Bcl-2/Bax ratios as well as increased 8-iso-PGF2α levels and apoptosis were observed (all P<0.05), which were effects that were attenuated by the treatment with NAC. NF-κBp65 and iNOS levels were significantly higher and the P-IκB-α levels were significantly lower in the HF group; expression of all three proteins returned to pre-HF levels following treatment with NAC. Myocardial cell apoptosis was positively correlated with left ventricular end-diastolic pressure (LVEDP), NF-κBp65 expression and 8-iso-PGF2α levels, but negatively correlated with the maximal and minimal rates of increase in left ventricular pressure (+dp/dtmax and −dp/dtmin, respectively) and the Bcl-2/Bax ratio (all P<0.001). The 8-iso-PGF2α levels were positively correlated with LVEDP and negatively correlated with +dp/dtmax and −dp/dtmin (all P<0.001). The present study demonstrated that NAC increased the antioxidant capacity, decreased the NF-κB activation and reduced myocardial cell apoptosis in an *in vivo* heart failure model.

## Introduction

Approximately 23 million people worldwide are estimated to have congestive heart failure (1), including 6.6 million Americans (2). Furthermore, the prevalence of heart failure is predicted to increase worldwide (3,4). A number of racial differences in the incidence of heart failure have been observed, including studies that revealed that although African-American patients are at a greatest risk of developing heart failure with subsequent hospitalization (5), the prevalence of atrial fibrillation in patients hospitalized with heart failure was higher in white patients (6). Oxidative stress has an important role in the occurrence and development of heart failure, which is characterized by contractile dysfunction (7). In patients with heart failure and *in vivo* models, excessive reactive oxygen species (ROS) production in the myocardium, accompanied by systemic inflammation, have been observed (8,9). Furthermore, it has been demonstrated that the level of oxidative stress is associated with the severity of heart failure and the grade of cardiac function (10).

Oxidative stress may induce myocardial cell apoptosis, resulting in cardiac tissue damage and the subsequent deterioration of hemodynamics (8,11). Inflammation-related nuclear factor (NF)-κB signaling and its correlation with apoptosis have been proposed as a mechanism underlying the pathogenesis of heart failure (12). Although a cardioprotective role for NF-κB in acute hypoxia has been observed, various studies have demonstrated that prolonged NF-κB activation induces myocardial injury (13,14). NF-κB is a transcription factor that regulates the expression of pro-inflammatory cytokines, including interleukin (IL)-1, IL-6 and tumor necrosis factor-α (TNF-α), as well as genes associated with apoptosis (e.g. p53) (14). In a previous study in NF-κB-null mice, improved cardiac function following myocardial infarction was observed (15). Oxidative stress may activate NF-κB and initiate the transcription of several pro-apoptotic genes, including *Bax*, *Fas* and *FasL*, inducing myocardial cell apoptosis and promoting heart failure.

Antioxidant therapy attenuates ischemia-reperfusion-induced apoptosis of cardiomyocytes (16). N-acetylcysteine (NAC), the precursor of glutathione (GSH), increases the intracellular content of GSH, stabilizes the cell membrane, protects the cellular viability and directly scavenges ROS (16). Thus, in ischemia-reperfusion injury, NAC is able to prevent ROS-induced apoptosis (17), and in ischemic heart failure, NAC reduced superoxide anion levels and restored cardiomyocyte contractility (18). The present study aimed to determine the effect of NAC on oxidative stress, myocardial apoptosis and NF-κB activation. An *in vivo* heart failure model was established in rabbits treated with doxorubicin, a chemotherapeutic agent with known dose-dependent cardiotoxicity, as previously described (19–21). The effect of NAC on myocardial apoptosis, NF-κB activation and expression, Bcl-2 and Bax expression, oxidative stress, inducible nitric oxide synthase (iNOS) expression and cardiac function was investigated. These studies will form the basis for further analysis of the therapeutic value of NAC in the treatment of heart failure.

## Materials and methods

### Establishment of an in vivo heart failure model

A total of 50 Japanese white big-ear rabbits were purchased from the Experimental Animal Center of Medicine College of Wuhan University (Wuhan, China). Ten rabbits served as controls (control group). Heart failure was induced by doxorubicin in the remaining 40 rabbits using previously described methods (19,22). Briefly, doxorubicin hydrochloride (Zhejiang HiSun Minsheng Pharmaceutical Co., Ltd, Zhejiang, China) was diluted in normal saline at a concentration of 1 mg/ml and then 1.0 mg/kg body weight was injected via the ear vein twice weekly for eight consecutive weeks. Heart failure was diagnosed by echocardiography with a sector scanning ultrasound probe at 8 MHz (GE Vivid VII color Doppler ultrasound, GE Medicals, Fairfield, CT, USA) at the end of eight weeks. Of the 25 rabbits that developed heart failure, 13 (NAC group) received 300 mg/kg NAC (Hangzhou Minsheng Pharmaceutical Co., Ltd, Hangzhou, Zhejiang, China) once daily for four weeks. The remaining 12 rabbits with heart failure (HF group) received normal saline of an equal volume. All of the animal experiments were approved by the Animal Care and Use Committee of Medicine College of Wuhan University.

### Echocardiography analysis

In all of the three groups, echocardiography was performed at the end of week 12 with a sector scanning ultrasound probe at 8 MHz (GE Vivid VII color Doppler ultrasound). Prior to the echocardiography, the animals received an intramuscular injection of diazepam (2 mg) for sedation. A parasternal long axis view of the left ventricle was used to detect the inner diameter of the left atrium and left ventricle, left ventricular end-diastolic diameter (LVEDD), left ventricular end-systolic diameter (LVESD) and interventricular septal thickness (IVST). The short axis view at the papillary muscle level was used for M-shaped sampling to detect the ejection fraction (EF) and fraction shortening (FS). The parasternal four-chamber view was used to observe the movement of the ventricular wall. The long-axis view of the pulmonary artery was employed to detect the inner diameter of the pulmonary artery and frequency spectrum. The apical three-chamber view, four-chamber view and five-chamber view were employed to detect the frequency spectrum of the aorta and mitral valve.

### Hemodynamics analysis and collection of myocardial tissue

At the end of the study, the rabbits in all groups were intravenously anesthetized with 20% urethane at 5 ml/kg. Following catheterization of the aorta, the heart rate (HR), left ventricular systolic pressure (LVSP), left ventricular end-diastolic pressure (LVEDP), peripheral mean arterial pressure (MAP), and the maximal and minimal rates of the rise in left ventricular pressure (+dp/dtmax and −dp/dtmin, respectively) were measured using the BL-420E biological function detection system (Chengdu Taimeng Science and Technology Co., Ltd, Chengdu, China). The animals were immediately sacrificed by injection of 5 ml of 10% potassium chloride. Thoracotomy was performed and the heart was collected. The left ventricle was isolated and fixed in 4% paraformaldehyde or liquid nitrogen for further use.

### Analysis of myocardial cell apoptosis

The myocardium was fixed in 4% paraformaldehyde, embedded in paraffin and sectioned. Terminal deoxynucleotidyl transferase-mediated dUTP nick end labeling (TUNEL) was performed using an *In Situ* Cell Death Detection kit (Roche, Mannheim, Germany) to detect the number of apoptotic cells according to manufacturer’s instructions. The normal cells were identified as having blue nuclei while the apoptotic cells had yellow-brown nuclei. Four sections were randomly selected from each rabbit, and five fields at a high magnification (x400) were randomly selected to count the number apoptotic myocardial cells and total myocardial cells. The apoptosis index (AI) was determined as the proportion of apoptotic cells relative to the total cells.

### Immunohistochemistry analysis of Bcl-2, Bax and NF-κBp65 expression

Immunohistochemistry analysis of NF-κBp65 was performed using a kit from Wuhan Boster Biotech Co., Ltd, Wuhan, China) according to the manufacturer’s instructions. The following primary antibodies diluted 1:100 were used: Anti-Bcl-2 (Wuhan Boster Biotech Co., Ltd.) and Bax (ZSGB-Bio, Beijing, China). Visualization was performed with DAB followed by counterstaining with hematoxylin and mounting with neutral gum. The tissues in which the primary antibody was replaced with phosphate-buffered saline (PBS) served as the negative control group. The cells positive for Bcl-2 or Bax had brown granules in the cytoplasm and on the cell membrane; the cells positive for NF-κB had brown granules in the nucleus. Five sections were selected from each group, and five fields were randomly selected at a high magnification (x400) for the detection of mean optical density using a HMIAS-2000 image analysis system (Guangzhou Longest Technology, Guangzhou, China). The optical density of Bcl-2, Bax and NF-κBp65 expression was obtained. Notably, as the target protein expression increased, the optical density decreased.

### Western blot analysis of NF-κBp65 and IκB-α expression

The myocardium was cut into pieces and 20 mg was mixed in 200 μl RIPA lysis buffer (50 mM Tris-HCl, pH 7.4; 150 mM NaCl and 1% NP-40) followed by homogenization (Lisure Science, Shanghai, China). Following centrifugation at 25,758 × g for 5 min, the supernatant was collected for the detection of protein concentration using the bicinchoninic acid method (Spectrum, Gardena, CA, USA). Aliquots of the supernatant were stored at −80°C. The proteins (20 μg) were separated by SDS-PAGE following which they were transferred onto a polyvinylidene difluoride membrane (Seebio, Shanghai, China). The membranes were blocked using 5% skimmed milk in 0.01 M PBS at room temperature for 2 h, following which they were incubated with the primary antibodies specific for NF-κBp65 (1:1000; Cell Signaling Technology, Inc., Beverly, MA, USA), IκB-α (1:2000; Wuhan Boster Biotech Co., Ltd) or β-actin (1:2000; Wuhan Boster Biotech Co., Ltd) overnight at 4°C. Following incubation with a horseradish peroxidase (HRP)-conjugated goat anti-rabbit antibody or HRP-conjugated goat anti-mouse antibody (1:2000; both from Jackson Immunoresearch, West Grove, PA, USA) at room temperature for 2 h, the bands were visualized using a chemiluminescent system (Wuhan Boster Biotech Co., Ltd). The gel image analysis system GelDoc- XR (Bio-Rad, Hercules, CA, USA) was used to semi-quantitatively detect the protein expression and normalize it to the β-actin values.

### Detection of total anti-oxidative capacity (tAOC) of serum and myocardium

Blood (3 ml) was collected from the common carotid artery prior to sacrifice followed by centrifugation at 2,191 × g for 15 min. The serum was collected and stored at −20°C until use. The left ventricle was weighed, cut into pieces and homogenized as a 10% myocardial homogenate. Following centrifugation at 179 × g for 10 min, the supernatant was collected for the detection of the tAOC of the serum and myocardium by colorimetry according to manufacturer’s instructions (Nanjing Jiancheng Biotech Co., Ltd, Nanjing, China) and as previously described (23). This measurement reflects the overall antioxidant status, including antioxidants yet to be identified (24). Briefly, 2,20-azino-di-(3-ethylbenzthiazoline-6-sulphonic acid) (ABTS) was incubated with peroxidase, metmyoglobin and H_2_O_2_, producing ABTS that was blue-green at 600 nm and colorless after it was reduced to ABTS in the presence of antioxidants (23). The change in color was reduced to a degree that was proportional to the antioxidant concentration. tAOC values were expressed as U/ml in serum samples and U/mg in myocardium.

### Detection of serum GSH

Blood (3 ml) was collected from the common carotid artery prior to sacrificing the animals and was centrifuged at 2,191 × g for 15 min. Following collection of the serum samples, the serum GSH levels were determined according to the manufacturer’s instructions (Nanjing Jiancheng Biotech Co., Ltd.).

### Detection of 8-iso-prostaglandin F2α by enzyme immunoassay (EIA)

At the end of the study and prior to sacrifice of the animals, venous blood (2 ml) was collected, and the serum was isolated by centrifugation at 2,862 × g for 15 min and stored at −80°C until use. The left ventricle was combined with PBS containing 0.1 mmol EDTA and homogenized. Following centrifugation at 2,862 × g for 15 min, the supernatant was collected for the detection of 8-iso-prostaglandin F2α (8-iso-PGF2α) by EIA following the manufacturer’s instructions (Cayman Chemical, Ann Arbor, MI, USA).

### Statistical analysis

Normally distributed continuous variables were compared by one-way analysis of variance. When a significant difference between the groups was apparent, multiple comparisons of means were performed using the Bonferroni procedure with type-I error adjustment. Data are presented as the mean ± standard deviation. The correlations between the apoptosis index/8-iso-PGF2α and cardiac function were examined using Pearson correlation coefficients. All of the statistical assessments were two-sided and P<0.05 was considered to indicate a statistically significant difference. Statistical analyses were performed using SPSS 15.0 statistics software (SPSS, Inc., Chicago, IL, USA).

## Results

### Effects of NAC on cardiac function and 8-iso-PGF2α levels

Cardiac function was assessed by echocardiography in the untreated, HF and NAC groups. As demonstrated in Table I, the LVEDD and LVESD were significantly higher, and the EF and FS were significantly lower in the HF group, as compared with the control group (P<0.001). However, treatment with NAC returned the LVEDD and LVESD to the control levels, and significant improvements in the EF and FS were also observed in the NAC group (P<0.001).

Cardiac function was also assessed by hemodynamic analysis. In the HF group, significantly lower MAP, LVSP, +dp/dtmax and −dp/dtmin levels were observed, as compared with the control groups (P<0.05), while the LVEDP was significantly higher (P<0.001; Table I). Following NAC treatment, the MAP, LVSP, LVEDP, +dp/dtmax and −dp/dtmin levels all returned to those observed in the control group (Table I). Thus, these results indicate that NAC significantly improved cardiac function in an *in vivo* model of heart failure.

### Effects of NAC on 8-iso-PGF2α levels

**Figure 1:**
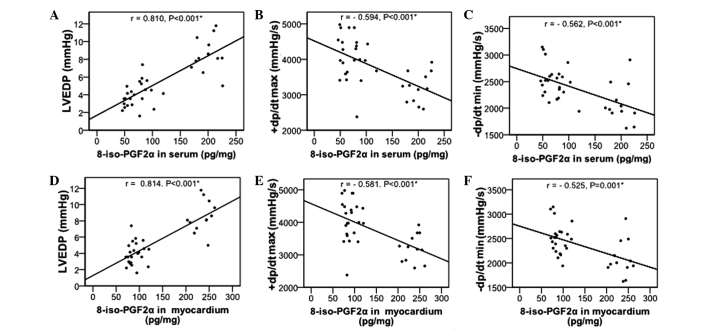
The correlation between 8-iso-PGF2α levels and cardiac function. The correlations were tested by determining Pearson correlation coefficients. 8-iso-PGF2α, 8-iso-prostaglandin F2α; LVEDP, left ventricular end-diastolic pressure; +dp/dtmax, maximal rate of rise of left ventricular pressure; −dp/dtmin, minimal rate of rise of left ventricular pressure.

It has been demonstrated that 8-iso-PGF2α may serve as a marker for myocardial injury and heart failure (25), its levels in the serum and myocardium were also determined. As revealed in Table II, significantly increased 8-iso-PGF2α levels in the serum and myocardium were observed in the HF group, as compared with the control group (P<0.05). NAC significantly decreased the 8-iso-PGF2α levels (P<0.01), but not to the levels observed in the control group. Furthermore, 8-iso-PGF2α levels in serum and myocardium were positively correlated with LVEDP and negatively correlated with +dp/dtmax and −dp/dtmin (Fig. 1; all P<0.001).

### NAC reduces oxidative stress in an in vivo model of heart failure

NAC increases the intracellular content of GSH and directly scavenges ROS (16), thus in the present study, its effects on serum and myocardial tAOC were determined to assess the level of oxidative stress. In addition, the serum GSH levels were measured in each treatment group. As demonstrated in Table II, the tAOC in the serum and myocardium was significantly lower in the HF group, as compared with the control group (P<0.05). Following the NAC treatment, tAOC returned to levels comparable with those of the control group. Similarly, serum GSH levels were markedly lower in the HF group, as compared with the control group (P<0.001). When compared with the HF group, the serum GSH level increased markedly in the NAC group (P<0.001) to levels comparable to those observed in the control group (Table II).

### Effects of NAC on myocardial cell apoptosis in heart failure

**Figure 2:**
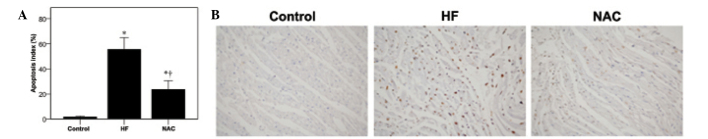
Effects of NAC on myocardial cell apoptosis in heart failure. (A) The apoptotic index was determined using the TUNEL assay. Pair-wise multiple comparisons between groups were determined using Bonferroni’s test with α=0.017 adjustment. ^*^P<0.05 indicates a statistically significant difference between the indicated group and the control group; ^†^P<0.05 indicates a statistically significant difference between the indicated group and the HF group. (B) Representative images of the TUNEL analysis from each group are demonstrated (magnification, ×400). NAC, N-acetylcysteine; HF group, untreated heart failure group; TUNEL, Terminal deoxynucleotidyl transferase-mediated dUTP nick end labeling.

NAC protects the cellular viability (16); therefore, its effects on myocardial cell apoptosis were determined using the TUNEL assay. As demonstrated in Fig. 2A, significantly increased levels of apoptosis was observed in the HF group as compared with the control group (1.57±0.88 vs. 55.62±9.35%, respectively; P<0.05). However, NAC treatment significantly reduced myocardial cell apoptosis (23.71±6.97%), but not to the control levels (P<0.001). The representative images of the TUNEL analysis from each group are shown in Fig. 2B. Specifically, the presence of yellow-brown granules and karyopyknosis was observed in the HF group (Fig. 2, middle panel), but not the control group (Fig. 2, left panel). Fewer TUNEL-positive nuclei were detected in the NAC group (Fig. 2, right panel).

**Figure 3:**
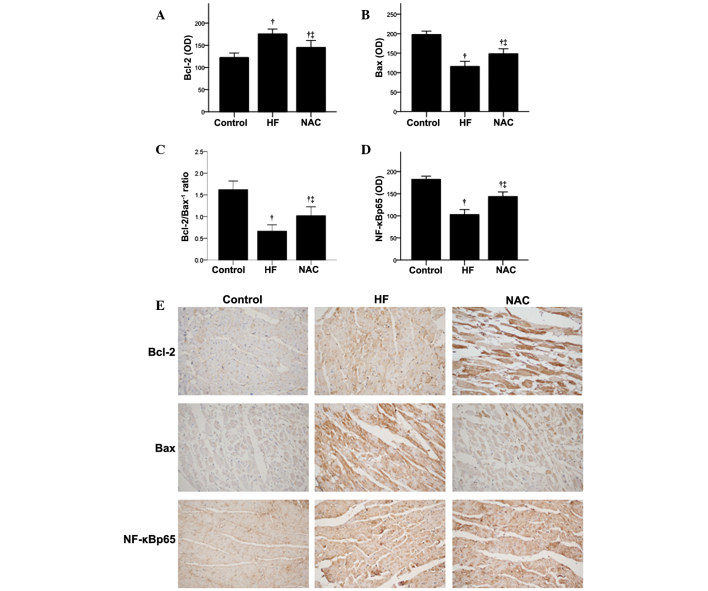
Effects of NAC on apoptosis-associated protein expression in heart failure. (A) Bcl-2, (B) Bax, (C) Bcl-2/Bax ratio and (D) NF-κBp65 protein expression was determined by immunohistochemical analysis. The mean OD was determined using an HMIAS-2000 image analysis system; the higher OD values indicate lower protein expression. P-values are based on analysis of variance and pair-wise multiple comparisons between groups were determined using Bonferroni’s test with α= 0.017 adjustment. ^†^P<0.05 indicates a significant difference between the indicated group and the control group; ^‡^P<0.05 indicates a significant difference between the indicated group and the HF group. (E) Representative images of Bcl-2 (top panels), Bax (middle panels) and NF-κBp65 (bottom panels) protein expression from each group are demonstrated (magnification, ×400). NAC, N-acetylcysteine; HF group, untreated heart failure group; NF-κB, nuclear factor κB; OD, optical density.

The expression of two apoptosis-related proteins, Bax and Bcl-2, were examined by immunohistochemistry (Fig. 3). In the HF group, Bax expression was significantly higher while Bcl-2 protein expression and the Bcl-2/Bax^−1^ ratio were significantly lower than that of the control group (P<0.05; Fig. 3A–C). In the NAC group, significantly decreased Bax protein expression and increased Bcl-2 and Bcl-2/Bax^−1^ ratio were observed, as compared with the HF group (P<0.05). These results suggest that NAC may improve cardiac function in heart failure by reducing cardiomyocyte apoptosis. Representative images of Bax and Bcl-2 protein expression reveal the absence of Bcl-2 and Bax expression in the control group (Fig. 3E). Bcl-2 immunoreaction was observed in the cytoplasm and on the cell membrane of a few myocytes in the HF group, as well as a variety of myocytes in the NAC group (Fig. 3E, top panels). Increased Bax immunoreaction was also observed in the cytoplasm and cell membrane of myocytes in the HF group, which was decreased in the NAC group (Fig. 3E, middle panels).

### Effects of NAC on NF-κBp65 expression and activity

**Figure 4:**
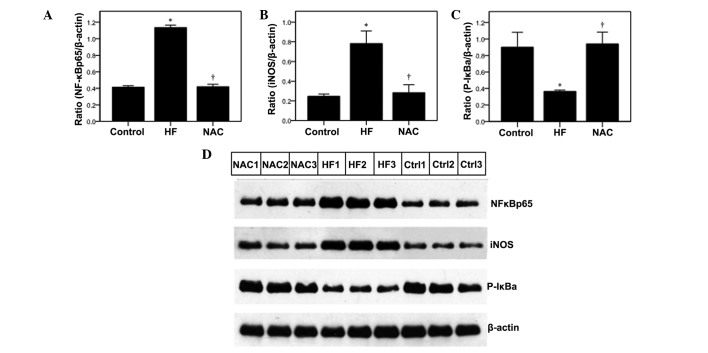
Effects of NAC on NF-κBp65 expression and activity. Relative (A) NF-κBp65, (B) iNOS and (C) P-IκB expression was determined using western blot analysis following normalization to β-actin. (D) Representative blots are demonstrated. Pair-wise multiple comparisons between groups were determined using Bonferroni’s test with α=0.017 adjustment. ^*^P<0.05 indicates a statistically significant difference between the indicated group and the control group; ^†^P<0.05 indicates a statistically significant difference between the indicated group and the HF group. NAC, N-acetylcysteine; HF group, untreated heart failure group; NF-κB, nuclear factor κB; iNOS, inducible nitric oxide synthase.

NF-κB-induced apoptosis has been associated with heart failure (12); therefore, the present study examined the NF-κBp65 expression using immunohistochemistry (Fig. 3D) and western blot analysis (Fig. 4). Immunohistochemistry analysis revealed that NF-κBp65 levels were significantly higher in the HF group than that observed for the control group (P<0.05), and NAC significantly decreased NF-κBp65 expression (P<0.05; Fig. 3D). The representative images of NF-κBp65 protein expression are demonstrated in Fig. 3E, which reveal diffuse cytoplasmic immunoreaction in the control group, with increased nuclear expression in the HF group. Reduced NF-κBp65-positive nuclei were observed in the NAC group. These results were confirmed using western blot analysis (Fig. 4).

The effects of NAC on NF-κBp65 activity were determined by measuring the phosphorylation of inhibitor κB (P-IκB) and its downstream target, inducible nitric oxide synthase (iNOS) (26), by western blot analysis. In the HF group, iNOS levels were significantly higher as compared with the control, which was reduced by NAC (Fig. 4B; P<v). In addition, P-IκB-α levels were significantly lower in the HF group, but increased to the control levels with NAC treatment (Fig. 4C).

### Correlation of myocardial cell apoptosis with cardiac function, NF-κBp65 and 8-iso-PGF2α

**Figure 5:**
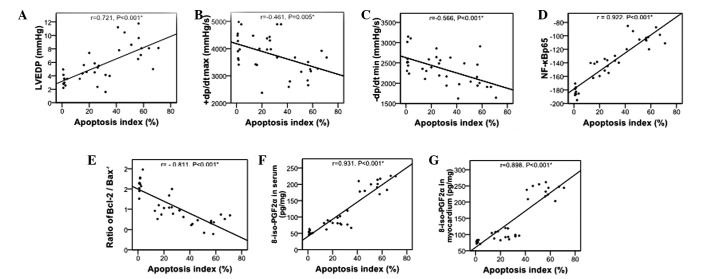
Correlation of myocardial cell apoptosis with cardiac function and expression of NF-κBp65 and 8-iso-PGF2α. The correlations were tested by determining Pearson correlation coefficients. The correlations of myocardial cell apoptosis index and (A) LVEDP; (B) +dp/dtmax; (C) −dp/dtmin; (D) NF-κBp65; (E) ratio of (Bcl-2/Bax)^−1^; (F) 8-iso-PGF2α in serum; and (G) 8-iso-PGF2α in myocardium. 8-iso-PGF2α, 8-iso-prostaglandin F2α; LVEDP, left ventricular end-diastolic pressure; +dp/dtmax, maximal rate of rise of left ventricular pressure; −dp/dtmin, minimal rate of rise of left ventricular pressure.

Apoptosis is a pathological feature of heart failure (12), its correlation with cardiac function, NF-κBp65 and 8-iso-PGF2α was assessed in the present *in vivo* model of heart failure (Fig. 5). Myocardial cell apoptosis was positively correlated with LVEDP (Fig. 5A), NF-κBp65 expression (Fig. 5D), and 8-iso-PGF2α levels in the serum and myocardium (Fig. 5F and G, respectively; all P<0.001). It was also negatively correlated with +dp/dtmax (Fig. 5B), −dp/dtmin (Fig. 5C) and Bcl-2/Bax^−1^ ratio (Fig. 5E; all P<0.001).

## Discussion

The effects of NAC on oxidative stress and NF-κB during heart failure were examined in the present study. Reduced cardiac function and tAOC, and increased 8-iso-PGF2α levels were verified in the HF group, which was attenuated with NAC treatment. The 8-iso-PGF2α levels were positively correlated with LVEDP and negatively correlated with +dp/dtmax and −dp/dtmin. In addition, NAC attenuated myocardial cell apoptosis and altered the Bcl-2/Bax ratio observed in the HF group. Furthermore, the increased NF-κBp65 and iNOS levels, and reduced P-IκB-α levels observed in the HF group were reversed by NAC treatment. Finally, myocardial cell apoptosis was positively correlated with LVEDP, NF-κBp65 expression and 8-iso-PGF2α levels, and negatively correlated with +dp/dtmax, −dp/dtmin and the Bcl-2/Bax ratio. Therefore, the level of myocardial apoptosis was closely associated with cardiac function, and ROS accumulation may represent an important precipitating factor for myocardial apoptosis, possibly through NF-κBp65 in heart failure.

Oxidative stress is a major mechanism underlying doxorubicin-induced heart failure, and endogenous ROS affects cardiac contractility (27). In the present study, decreased serum, and myocardial tAOC and GSH levels were observed with the induction of heart failure, and these effects were reversed by NAC. This is consistent with a previous study by Finn and Kemp (28), which proposed that NAC alters GSH levels by pro-oxidant and antioxidant mechanisms. Although antioxidant and pro-oxidant effects of NAC and GSH have been previously reported (29), the present study demonstrated according to the tAOC values that NAC acts as an antioxidant.

Plasma 8-iso-PGF2α content increases significantly in patients with cardiovascular disease (25). The 8-iso-PGF2α levels reflect the severity of heart failure (on the basis of New York Heart Association classification) (30), but not the left ventricular ejection fraction (25). Therefore, 8-iso-PGF2α may serve as a marker for myocardial injury and heart failure. In the present study, 8-iso-PGF2α levels increased in the serum and myocardium of rabbits with doxorubicin-induced heart failure. Furthermore, the 8-iso-PGF2α levels were correlated with cardiac function (i.e., LVEDP and ±dp/dtmax), which is consistent with its function as a putative marker of heart failure.

Lipid peroxidation and calcium overload may induce oxidative stress and the accumulation of ROS (31), and result in myocardial cell apoptosis. In the present study, the severity of myocardial apoptosis was closely associated with the cardiac function. Overproduction of ROS may also stimulate the expression of certain apoptosis-associated genes, including Fas, Bcl-2, Bax and p53, inducing myocardial cell apoptosis (10,32). In the present study, increased myocardial cell apoptosis and expression of the pro-apoptotic protein, Bax, was observed in the HF group, that coincided with reduced Bcl-2 expression, and these effects were reversed by NAC. This result is consistent with those of previous studies describing the role of oxidative stress-induced myocardial apoptosis in the occurrence and development of heart failure (12,33).

In the present study, TUNEL analysis was used to assess the level of myocardial cell apoptosis in each group; however, this assay also detects DNA breaks induced by oxidative stress. Although the changes in the levels of apoptosis-associated proteins were consistent with induction of myocardial apoptosis and heart failure, further studies may use other assays to measure the extent of apoptosis, including determining caspase activation and trypan blue and propidium iodide exclusion assays. In addition, the presence of apoptotic myocardial cells in the HF group eight weeks following doxorubicin exposure suggests that this model is more representative of an ongoing induction of cardiomyopathy rather than established heart failure. This observation is consistent with those of previous studies (20,21). Specifically, in addition to the acute and chronic side effects associated with doxorubicin treatment, delayed toxicity (including ventricular dysfunction, heart failure and arrhythmias) has been observed decades after discontinuation of treatment and may be mediated by impaired sarcoplasmic reticulum calcium storage, DNA lesions induced by free radicals and reduced regenerative capacity (20). Recent *in vivo* data in mice suggest that long-term cardiac injury associated with doxorubicin may be reduced with aerobic exercise as well as resveratrol supplementation (21). However, further clinical studies are required to verify these protective effects in patients with doxorubicin-induced cardiomyopathy.

Increased NF-κB activity has been observed in an *in vivo* chronic stress model (13), and its inhibition protected against ischemia-reperfusion injury (34,35). IκB maintains NF-κB in an inactive state sequestered in the cytoplasm. Extracellular stimuli, including cytokines and oxidative stress, may result in IκB phosphorylation and subsequent dissociation from NF-κB. NF-κB then rapidly translocates into the nucleus, binding specific elements in the promoters of target genes and initiating their transcription (25,36). NF-κB also has an important role in oxidative stress-induced apoptosis. In heart failure, NF-κB initiated the expression of pro-apoptotic genes, including Bax and Fas, which induced myocardial and endothelial cell apoptosis (37). In the present study, NF-κBp65 expression and activity increased with heart failure and this increase was reduced following treatment with NAC. In addition, NF-κBp65 expression was positively correlated with the extent of myocardial apoptosis. This is consistent with the results of Maier *et al* (38), who induced cardiomyopathy and heart failure through IκB kinase (IKK)/NF-κB signaling. These results suggest that overproduction of ROS may induce NF-κB activation; however, its specific role in oxidative stress-induced myocardial apoptosis requires additional analysis.

Upon phosphorylation, IκB-α is ubiquitinated and subsequently subject to proteasome-mediated degradation (39). In the present study, P-IκB-α levels were significantly lower in the HF group and were attenuated with NAC. It is possible that the decrease in P-IκB in the HF model is a result of the proteasomal degradation of P-IκB. This would be consistent with a study by Pye *et al* (40) in which NF-κB activity was inhibited by a 20S proteasome inhibitor in an *in vivo* model of myocardial reperfusion injury, possibly through the inhibition of IκB degradation and NF-κB nuclear translocation (41).

NAC increases intracellular GSH levels, which stabilizes the cell membrane and prevents apoptosis. In ischemia-reperfusion-induced injury, NAC may scavenge ROS, preventing the induction of apoptosis (42). In addition, NAC restores cardiomyocyte contractility (18,27) and may protect against anthracyline cardiotoxicity (19). NAC may also inhibit NF-κB activity as was observed previously in leukemic cells (28), thereby suppressing the release of pro-inflammatory cytokines, including IL-8 and TNF-α. In the present study, treatment with NAC for eight weeks increased the tAOC and the Bcl-2/Bax ratio, and reduced the levels of myocardial cell apoptosis and NF-κBp65 expression, culminating in improved cardiac function, as is consistent with the results of Crespo *et al* (43). This suggests that anti-oxidative therapy may improve cardiac function via inhibiting apoptosis. NAC may inhibit oxidative stress by directly scavenging ROS (16), thus increasing the tAOC. Furthermore, NAC decreased isoproterenol-induced cardiotoxicity through its ROS scavenging, thereby reducing lipid hydroperoxide and 8-isoprostane levels (44), as well as the mitochondrial enzyme and calcium levels (45). Furthermore, NAC may inhibit NF-κB-mediated expression of pro-inflammatory cytokines and apoptosis-associated genes as was observed in an *in vivo* study of heart failure, in which the inhibition of TNF-α-related signal transduction by NAC promoted the recovery of myocardial structure and function (46).

In the present study, NAC increased the antioxidant capacity, decreased NF-κB activation and reduced myocardial cell apoptosis in an *in vivo* heart failure model. These results are consistent with those previously reported in rodent models (47,48). Specifically, NAC reduced *in vivo* cardiomyocyte dysfunction induced by behavioral stress, in part through modulating intracellular calcium signaling; however, the effects of NAC were independent of changes in GSH (47). In diabetic rats, NAC reduced myocardial reperfusion injury through increasing adiponectin levels and adiponectin receptor 2 expression, and restoring endothelial nitric oxide synthase activation (48). However, clinical studies indicate that the effects of NAC in preventing anthracycline-induced cardiomyopathy is limited (49,50). In a prospective randomized study of 19 patients with doxorubicin-induced cardiomyopathy, Dresdale *et al* (49) reported no difference in the LV ejection fraction (LVEF) or clinical course of the disease with NAC treatment. In another prospective randomized study of 103 Korean patients with breast cancer or lymphoma, NAC did not improve the observed reductions in LVEF in anthracycline-induced cardiomyopathy (50). These studies are however, limited in their size, so future clinical studies with higher NAC doses or longer duration may prove NAC to be more efficacious.

The present study is limited in that the direct effects of NAC were not assessed. In addition, the effects of ROS on other signaling pathways (e.g., SAPK, JNK and p38 signaling pathways) beyond NF-κB were not determined. Furthermore, while tAOC and GSH levels were determined, the enzymatic antioxidant capacity (e.g., superoxide dismutase, catalase and glutathione peroxidase) was not assessed.

In conclusion, NAC may inhibit oxidative stress, suppress NF-κB activation and regulate the expression of apoptosis-associated genes, such as Bax and Bcl-2, which may in turn reduce myocardial cell apoptosis and inflammation, and improve cardiac function in heart failure. Further studies are required to elucidate the mechanism underlying the effects of NAC, as well as its therapeutic value in the treatment of heart failure.

## Figures and Tables

**Table I:** Analysis of cardiac function in heart failure and after treatment with NAC.

	Control group (n=10)	HF group (n=12)	NAC group (n=13)	P-value
Cardiac echocardiography				
LVEDD (mm)	12.0±1.1	16.1±2.0^a^	12.5±1.1^b^	<0.001
LVESD (mm)	7.2±0.6	12.6±1.0^a^	8.3±1.2^b^	<0.001
IVST(mm)	1.8±0.3	1.8±0.3	1.9±0.3	0.698
EF (%)	72.5±9.7	42.3±8.3^a^	61.9±6.7^a^,^b^	<0.001
FS (%)	40.2±4.9	20.9±2.8^a^	34.0±5.0^a^,^b^	<0.001
Hemodynamics				
HR (beat/ min)	282.4±7.3	277.4±11.8	284.8±15.7	0.339
MAP (mmHg)	95.6±11.6	82.5±10.4^a^	90.5±10.9^b^	0.027
LVSP (mmHg)	109.7±6.3	95.1±10.1^a^	106.1±5.4^b^	<0.001
LVEDP (mmHg)	3.3±0.8	8.5±2.0^a^	4.5±1.5^b^	<0.001
+dp/dt (mmHg/s)	4169±550	3208±430^a^	4014±687^b^	0.001
−dp/dt (mmHg/s)	2640±330	2088±369^a^	2510±169^b^	<0.001

P-values are based on an analysis of variance test. Pair-wise multiple comparisons between groups were determined using Bonferroni’s test with α=0.017 adjustment.

a P<0.05 between the indicated group and the control group;

b P<0.05 between the indicated group and the HF group.

NAC, N-acetylcysteine; HF group, untreated heart failure group; LVEDD, left ventricular end-diastolic diameter; LVESD, left ventricular end-systolic diameter; IVST, interventricular septal thickness; EF, ejection fraction; FS, fraction shortening; HR, heart rate; MAP, peripheral mean arterial pressure; LVSP, left ventricular systolic pressure; LVEDP, left ventricular end-diastolic pressure; +dp/dtmax, maximal rate of rise of left ventricular pressure; −dp/dtmin, minimal rate of rise of left ventricular pressure.

**Table II:** Effects of NAC on tAOC and 8-iso-PGF2α in serum and myocardium among the groups.

	Control group (n=10)	HF group (n=12)	NAC group (n=13)	P-value
tAOC				
Serum (U/ml)	15.09±4.03	8.86±2.21^a^	13.23±2.92^b^	<0.001
Myocardium (U/mg)	1.65±0.20	1.26±0.30^a^	1.58±0.19^b^	0.001
8-iso-PGF2α				
Serum (pg/mg)	53.22±5.33	199.58±19.16^a^	85.01±15.12^a^,^b^	<0.001
Myocardium (pg/mg)	78.08±4.41	235.49±18.52^a^	99.48±12.16^a^,^b^	<0.001
GSH (unit/ml)	28.18±2.58	12.95±2.87^a^	22.39±2.75^a^,^b^	<0.001

P-values are based on analysis of variance test. Pair-wise multiple comparisons between groups were determined using Bonferroni’s test with α=0.017 adjustment.

a P<0.05 between the indicated group and the control group;

b P<0.05 between the indicated group and the HF group.

NAC, N-acetylcysteine; HF group, untreated heart failure group; tAOC, total anti-oxidative capacity; 8-iso-PGF2α 8-iso-prostaglandin F2α; GSH, glutathione.
